# Supplementary figures and images for: Opposing regulation of TNF responses by IFN-γ and a PGE2-cAMP axis that is apparent in rheumatoid and immune checkpoint inhibitor-induced arthritis human IL-1β+ macrophages
Source: eLife. 2025 Jul 15;14:RP104367. doi: 10.7554/eLife.104367 (PMC12263154; doi:10.7554/eLife.104367)

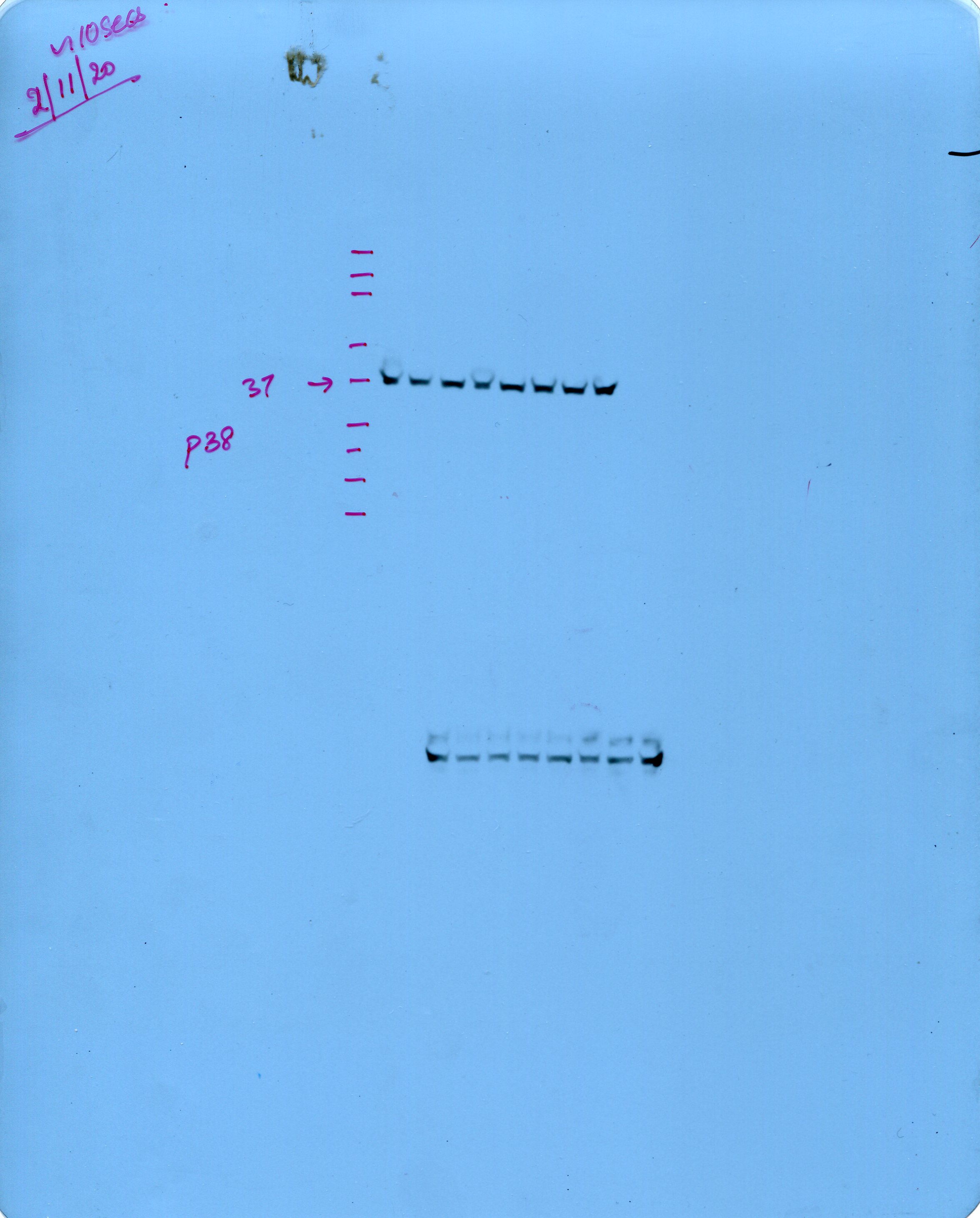

Supplement: Figure 1—source data 1. [file elife-104367-fig1-data1.zip › For upload of source data/p38 blot Fig 1F.jpg]

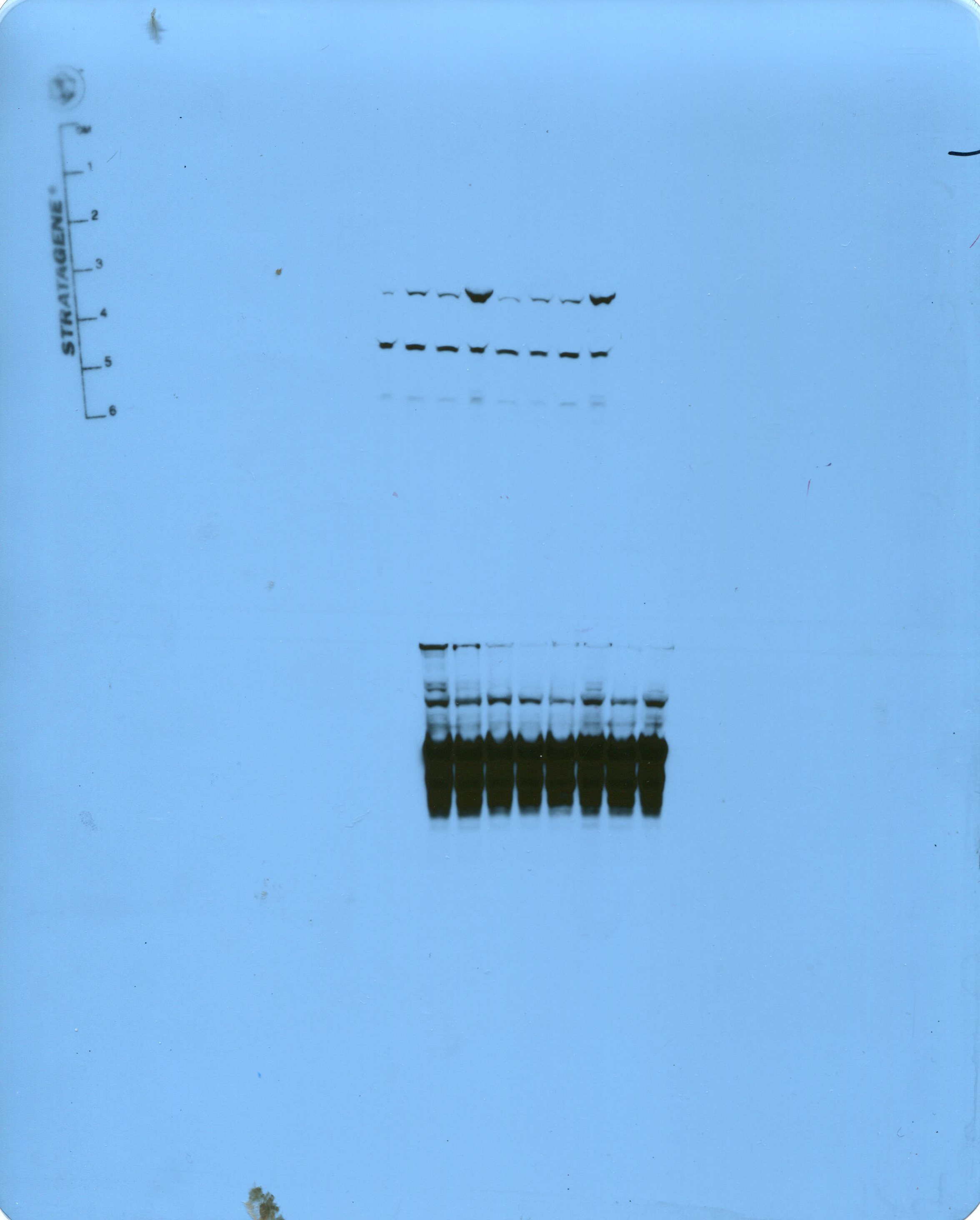

Supplement: Figure 1—source data 1. [file elife-104367-fig1-data1.zip › For upload of source data/Stat4 blot Fig 1F exposure 1.jpg]

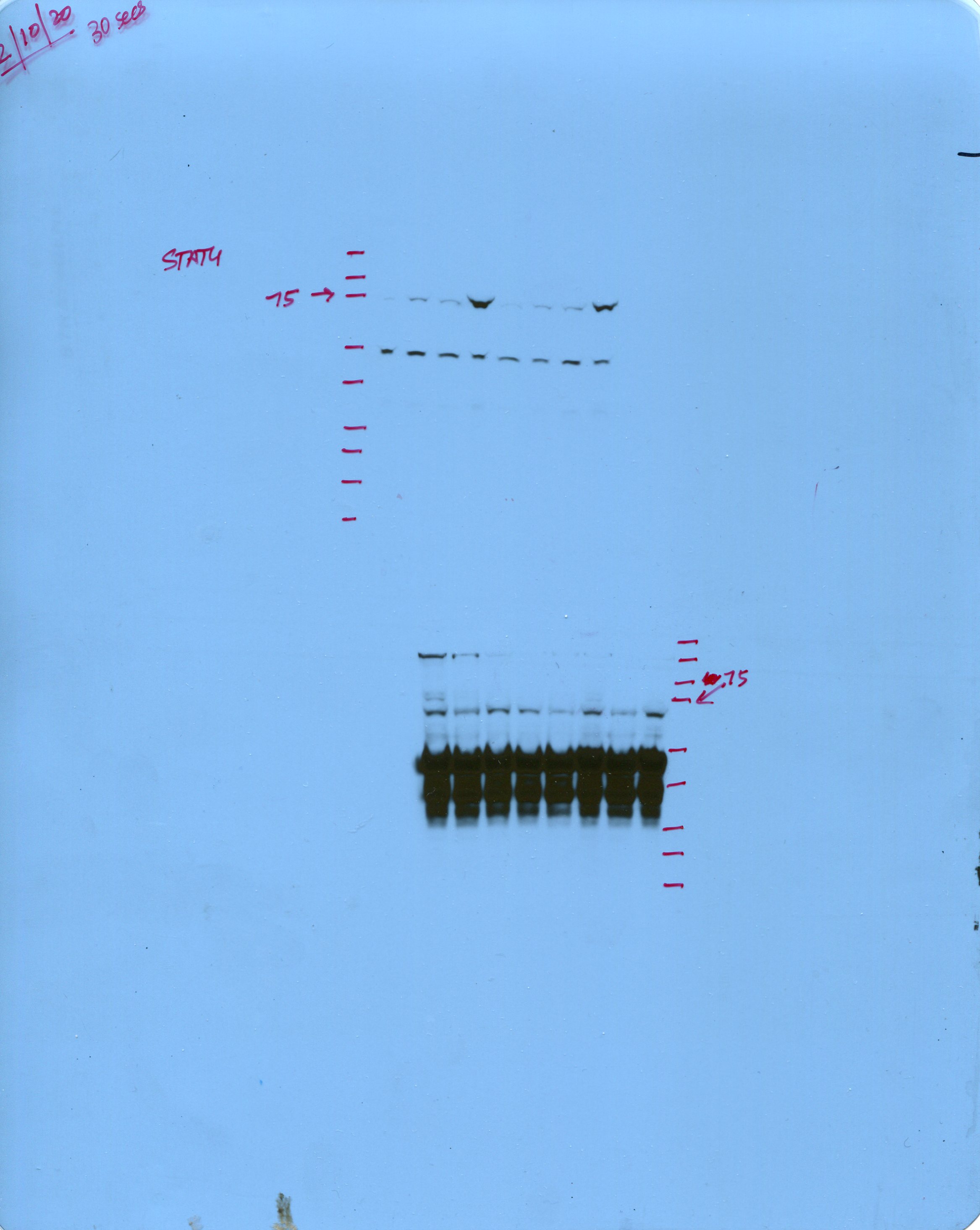

Supplement: Figure 1—source data 1. [file elife-104367-fig1-data1.zip › For upload of source data/Stat4 blot Fig 1F exposure 2.jpg]
