# Supplementary material for: Opposing regulation of TNF responses by IFN-γ and a PGE2-cAMP axis that is apparent in rheumatoid and immune checkpoint inhibitor-induced arthritis human IL-1β+ macrophages
Source: eLife. 2025 Jul 15;14:RP104367. doi: 10.7554/eLife.104367 (PMC12263154; doi:10.7554/eLife.104367)
Supplement: Figure 1—source data 2. [file elife-104367-fig1-data2.zip › Labeled PDF source data/Labelled source data Fig 1F.pdf]

Stat4 blot exposure 1

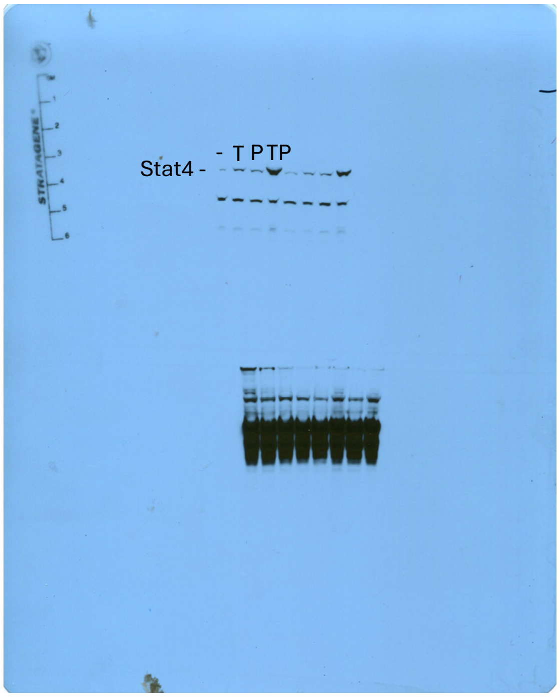

Stat4 blot exposure 2

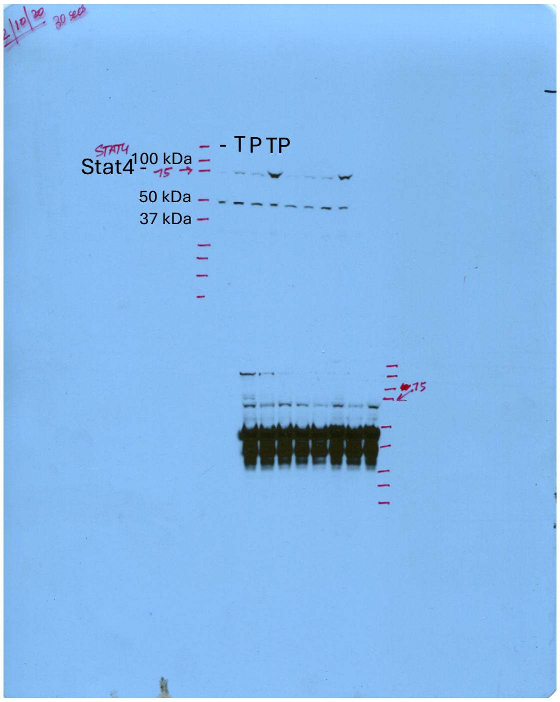

p38 blot

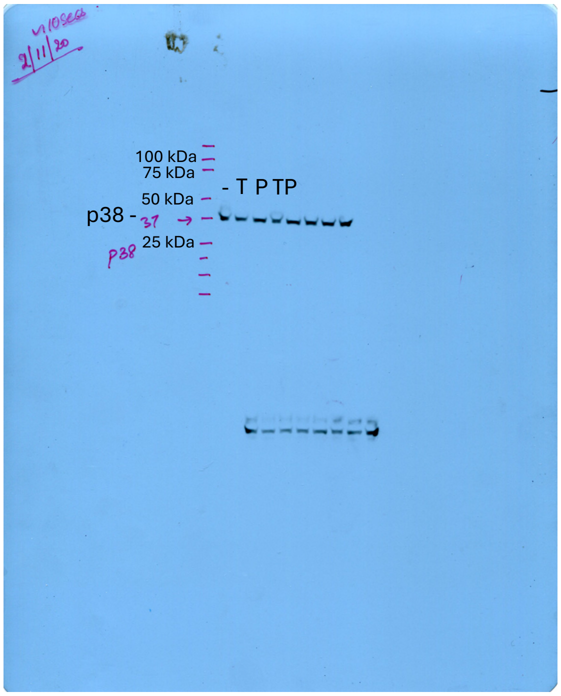

Figure 1F, source data 2. PDF file containing original western blots indicating relevant bands and treatments. The red writing was on original film and the black text is additional labelling. Stat4 blot exposure 1 was used in Figure 1F; exposure 2 shows original labeling of bands and MW markers. p38 was used as a loading control. Lanes 1-4 were used in Figure 1F.
- = control; T = TNF; P = PGE2; TP = TNF + PGE2
