# Supplementary material for: Opposing regulation of TNF responses by IFN-γ and a PGE2-cAMP axis that is apparent in rheumatoid and immune checkpoint inhibitor-induced arthritis human IL-1β+ macrophages
Source: eLife. 2025 Jul 15;14:RP104367. doi: 10.7554/eLife.104367 (PMC12263154; doi:10.7554/eLife.104367)
Supplement: Supplementary file 1. [file elife-104367-supp1.docx]

Supplementary File 1: Clinical characteristics of ICI-arthritis patients

| **Pt ID** | **Age** | **Sex** | **Primary Malignancy** | **ICB-therapy** | **Arthritis Pattern** | **ICB Initiation to arthritis Onset**  (wks) | **Arthritis Onset to SF Collection**  (wks) | **ICB Initiation to SF Collection**  (wks) | **Most Recent ICB to SF Collection**  (wks) | **Duration of ICI- therapy** | **CDAI** |
| --- | --- | --- | --- | --- | --- | --- | --- | --- | --- | --- | --- |
| **HSS101** | 65 | M | Melanoma | PD-1 | Polyarthritis | 4.3 | 147.6 | 151.9 | 106.6 | 46.3 | 34.0 |
| **HSS110** | 50 | F | Melanoma | PD-1 + CTLA-4 | Polyarthritis | 52.3 | 173.9 | 226.2 | 185.0 | 42.2 | 9.0 |
| **HSS180** | 57 | F | Renal cell carcinoma | PD-1 + CTLA-4 | Monoarthritis | 40.1 | 0.7 | 40.8 | 17.4 | 24.4 | 12.0 |
| **HSS200** | 62 | F | Urothelial carcinoma | PD-1 | Oligoarthritis | 27.7 | 8.6 | 36.3 | 1.5 | 35.8 | 9.0 |
| **HSS219** | 55 | M | Urothelial carcinoma | PD-1 | Monoarthritis | 269.7 | 21.9 | 291.6 | 32.6 | 260.0 | 7.0 |
| **RA335L and RA335R** | 60 | **F** | Melanoma | PD-1 | Polyarthritis | 182.0 | N/A | N/A | N/A | N/A | N/A |
